# High Burden of Coinfections With Epidemic-Prone Pathogens Among Febrile Patients in Nigeria: A Multi-Pathogen Surveillance Study

**DOI:** 10.1093/cid/ciaf516

**Published:** 2025-11-20

**Authors:** Cyril Erameh, Lauren P Courtney, Vivian Kwaghe, Jay Osi Samuels, Claire A Quiner, Jean H Kim, Osahogie Isaac Edeawe, Nankpah Godsave Vongdip, Adamu Zigwai Ephraim, Onyia Justus Ejike, Ikponmwosa Odia, Katherine Asman, Philippe Chebu, Jacqueline Agbukor, Oladimeji Damilare Matthew, Victoria Orok, Femi Owolagba, Blessed Okhiria, Ephraim Ogbaini-Emovon, Walter Mary Odion, Blessing Amierhobhiye Obagho, Richard Fayomade, Emmanuel A Oga

**Affiliations:** Institute of Viral and Emergent Pathogens Control and Research, Irrua Specialist Teaching Hospital, Edo, Nigeria; Solutions, RTI International, Durham, North Carolina, USA; Internal Medicine, University of Abuja Teaching Hospital, Abuja, Nigeria; Laboratory Services, APIN Public Health Initiatives, Federal Capital Territory, Nigeria; Solutions, RTI International, Durham, North Carolina, USA; Solutions, RTI International, Durham, North Carolina, USA; Institute of Viral and Emergent Pathogens Control and Research, Irrua Specialist Teaching Hospital, Edo, Nigeria; Internal Medicine, University of Abuja Teaching Hospital, Abuja, Nigeria; Solutions, RTI International, Durham, North Carolina, USA; Internal Medicine, University of Abuja Teaching Hospital, Abuja, Nigeria; Institute of Viral and Emergent Pathogens Control and Research, Irrua Specialist Teaching Hospital, Edo, Nigeria; Solutions, RTI International, Durham, North Carolina, USA; Laboratory Services, APIN Public Health Initiatives, Federal Capital Territory, Nigeria; Institute of Viral and Emergent Pathogens Control and Research, Irrua Specialist Teaching Hospital, Edo, Nigeria; Internal Medicine, University of Abuja Teaching Hospital, Abuja, Nigeria; Internal Medicine, University of Abuja Teaching Hospital, Abuja, Nigeria; Laboratory Services, APIN Public Health Initiatives, Federal Capital Territory, Nigeria; Internal Medicine, University of Abuja Teaching Hospital, Abuja, Nigeria; Institute of Viral and Emergent Pathogens Control and Research, Irrua Specialist Teaching Hospital, Edo, Nigeria; Institute of Viral and Emergent Pathogens Control and Research, Irrua Specialist Teaching Hospital, Edo, Nigeria; Institute of Viral and Emergent Pathogens Control and Research, Irrua Specialist Teaching Hospital, Edo, Nigeria; Laboratory Services, APIN Public Health Initiatives, Federal Capital Territory, Nigeria; Solutions, RTI International, Durham, North Carolina, USA; ClineEpi Partners, Columbia, Maryland, USA

**Keywords:** acute febrile illness surveillance; coinfections; infectious disease epidemiology; infectious disease surveillance

## Abstract

**Background:**

Characterizing the etiology of acute febrile illness (AFI) remains a critical public health priority in malaria-endemic regions such as Nigeria, where constrained diagnostic capacity frequently contributes to misdiagnosis and delays in outbreak detection and response. This study aimed to explore the burden and nature of pathogen coinfections among febrile patients and to assess whether coinfection patterns were associated with clinical or epidemiologic differences.

**Methods:**

Patients presenting with AFI at tertiary hospitals in north-central and southern Nigeria were screened for 25 pathogens using the TaqMan Array Card multi-pathogen polymerase chain reaction. Enrollment was conducted over a 12-month period at each site, August 2023 to September 2024. The analysis focused on the pathogen distribution in coinfections and any associated outcomes for coinfected patients.

**Results:**

Among the 1200 participants enrolled, 20 pathogens were detected in 57.9% of enrollees. Coinfections were detected in 12.6%. Among them, 2 pathogens were detected in 36.0% and 3 or 4 pathogens were detected in 1.2%. Nearly all coinfections included either *Plasmodium* or *Rickettsia*. Notably, coinfected patients were not clinically distinguishable from those with single-pathogen infections based on severity of the illness, presence of underlying health conditions, or risk behaviors such as use of mosquito nets. Among those with viral hemorrhagic fevers, those with coinfections exhibited a notably higher case fatality rate (11.5%) than those with single-pathogen infections (4.2%).

**Conclusions:**

Given the challenge of clinically distinguishing between infections with overlapping symptomology, there is an urgent need for multi-pathogen diagnostics for clinical and public health use.

## Teaser

This study used a multi-pathogen TaqMan Array Cards to screen for 25 pathogens in patients with acute undifferentiated fever in Nigeria. Frequent coinfections were revealed, highlighting the diagnostic challenges of relying solely on syndromic approaches. Understanding the presence of coinfections is critical for both clinicians and public health decision-makers in determining underlying causes of illness.

Understanding the causes of acute febrile illness (AFI) is a critical public health concern, especially in malaria-endemic regions like Nigeria, where limited diagnostic capacity often leads to misdiagnosis and delayed outbreak detection and response. The presence of coinfections complicates diagnosis and treatment, especially in cases of AFI. Additionally, undiagnosed coinfections elicit public health concern because they could include an undiagnosed case of a pathogen of epidemic potential.

Coinfections have been detected in AFI patients, especially in sub-Saharan Africa and regions with high endemicity of malaria and other infectious diseases [1]. Coinfections involving *Plasmodium* have been extensively documented in the literature, including concurrent infections with *Salmonella* spp. (typhoid fever) [2], arboviruses such as dengue and chikungunya [3], and intestinal helminths like *Schistosoma* and filarial parasites [4].

Several pathogens of public health importance are endemic to Nigeria, in addition to *Plasmodium*. Lassa fever, a viral hemorrhagic fever caused by the Lassa virus, is endemic in several states and has caused recurrent outbreaks with high case fatality rates. Similarly, arboviral diseases such as chikungunya and dengue have reemerged as threats globally [5]. In addition to viral pathogens, bacterial zoonoses such as leptospirosis and rickettsioses are increasingly recognized causes of AFI [6, 7], highlighting the need for improved diagnostic and surveillance systems.

Patients are often overdiagnosed for malaria or other common diseases. The tendency to diagnose malaria frequently results in the oversight of critical pathogens because of their similar symptomology. Even if a common pathogen, such as malaria, is accurately detected by hospital laboratory tests, coinfections are not considered and will be overlooked. It is unknown how many coinfections may be overlooked or how frequently coinfections occur. This diagnostic gap underscores the need for broader screening tools. Unlike traditional clinical or gold standard assays, which are limited in the number of pathogens they can detect per specimen, multi-pathogen platforms such as Thermo Fisher's TaqMan Array Cards (TACs) enable simultaneous detection of dozens of pathogens. Although TAC is a research use–only tool and results require clinical confirmation, it offers a valuable approach for uncovering potential causes of AFI—particularly in settings where malaria overdiagnosis is common and diagnostic capacity is constrained.

Multi-pathogen surveillance research has emerged in recent years with the advancement of laboratory tools such as TAC, enabling detection of over 50 molecular targets. A multi-pathogen study in Tanzania used the TAC platform to detect coinfections in 50% of their study population and coinfections with 4–7 pathogens in 25% of the population, including viral, bacterial, and parasitic agents [1]. However, current surveillance research in sub-Saharan Africa is limited and insufficient for the detection of the diversity of endemic pathogens. Many of the surveillance studies conducted in the region are pathogen-specific and often target malaria, but other AFI-causing pathogens are often excluded [1, 2, 8, 9].

In settings where AFI is highly prevalent but diagnostic resources are constrained, uncertainty around disease etiology remains a persistent public health challenge. Clinicians frequently diagnose malaria by default, potentially overlooking other pathogens with similar symptoms—including those with epidemic or emerging potential. While single-pathogen surveillance systems dominate global health infrastructure, the true spectrum of febrile illness, including coinfections, remains poorly characterized. To address this gap, this study employed a broad molecular screening platform to investigate the diversity and frequency of pathogen coinfections in 2 Nigerian referral hospitals. By identifying up to 25 pathogens that may be overlooked in routine surveillance and clinical practice, we aimed to inform future biosurveillance, outbreak preparedness, and potentially clinical decision-making. Further, the study characterizes the burden, composition, and clinical correlates of coinfections across bacterial, viral, and protozoan pathogens among patients presenting with AFI.

## METHODS

A comprehensive description of the methods, procedures, and details of surveillance of AFI etiologies in Nigeria (SAFIAN) study can be found in [10]. In brief, SAFIAN was conducted in 2 hospitals in Nigeria-Irrua Specialist Teaching Hospital in Edo State and University of Abuja Teaching Hospital in the Federal Capital Territory (FCT). Participants were recruited from August 2023 to September 2024, spanning 12 months. Local staff identified patients at each hospital, and they were enrolled in SAFIAN if they met the criteria for AFI: (1) a measured temperature of ≥37.5°C or a history of fever within the preceding 10 days and (2) age ≥5 years.

After patient eligibility was confirmed and consent/assent was given, participants were enrolled and given a ₦4000 incentive. A phlebotomist collected blood and serum samples. Demographic, risk factor, clinical, treatment, and diagnostic data were collected and managed using standardized REDCap electronic forms. Blood samples were analyzed for 25 molecular targets using TACs and supported by extensive quality control protocols, as shown in Table 1 [10]. Lassa virus testing was also performed using the RealStar Lassa Virus RT-PCR kit (Altona Diagnostics; Hamburg, Germany).

**Table 1.** List of Molecular Targets Included on Customized SAFIAN TAC

Pathogen Type	Target	Abbreviation
Bacterial		
	*Bartonella* spp.	BART
	*Brucella* spp.	BRUC
	*Coxiella burnetii*	CBUR
	*Leptospira* spp.	LEPT
	*Neisseria meningitidis*	NMEN
	*Rickettsia* spp.	RICK
	Pan-*Salmonella*	pSALM
	*Yersinia pestis*	YPES
Protozoan		
	*Leishmania* spp.	LEISH
	*Plasmodium* spp.	PLAS
	*Trypanosoma brucei*	TBRU
Viral		
	Crimean-Congo hemorrhagic fever virus	CCHFV
	Chikungunya virus	CHIKV
	Dengue virus	DENV
	Hepatitis E virus	HEV
	Hantavirus	HTNV
	Lassa virus	LASV
	Marburg/Ebola virus	MARV/EBOV
	Monkeypox virus	MPOX
	O’nyong’nyong virus	ONNV
	Pan-*Orthopoxvirus*	pOPXV
	Rift Valley fever virus	RVFV
	West Nile virus	WNV
	Yellow fever virus	YFV
	Zika virus	ZIKV

Data integration was conducted using R Studio 2023.06.0 (Boston, MA). Analyses to examine prevalence, demographic distributions, and associations between pathogen detection and variables through χ² tests were performed using SAS 9.4 (Cary, NC).

## RESULTS

### Pathogen Distribution

One or more pathogens were detected in 57.9% (*n* = 695) of the 1200 participants enrolled, with coinfections identified in 12.6% (*n* = 152) of them (Table 2). Of the participants with pathogens detected, 36.0% (*n* = 152 of 695) were coinfected with multiple pathogens and 1.2% (*n* = 8 of 695) were found to have 3 or 4 pathogens.

**Table 2.** Number of SAFIAN Participants With Pathogens Detected, Total, by Site and by Age Group

Pathogen Detected by SAFIAN Study	No. of Participants (%) (*n* = 1200)	Edo (%) (*n* = 600)	FCT (%) (*n* = 600)	Adult (%) (*n* = 750)	Child (%) (*n* = 450)
One or more pathogens detected	695 (57.9)	403 (67.2)	292 (48.7)	411 (54.8)	284 (63.1)
Single pathogen detected	543 (45.3)	311 (51.8)	232 (38.7)	319 (42.5)	224 (49.8)
Coinfection detected with 2 pathogens	143 (11.9)	84 (14.0)	59 (9.8)	84 (11.2)	59 (13.1)
Coinfection detected with 3 pathogens	8 (0.7)	7 (1.2)	1 (0.2)	7 (0.9)	1 (0.2)
Coinfection detected with 4 pathogens	1 (0.1)	1 (0.2)	—	1 (0.1)	—
No pathogens detected	505 (42.1)	197 (32.8)	308 (51.3)	339 (45.2)	166 (36.9)

A higher number of participants from Edo State (*n* = 403; 67.2%) had 1 or more pathogens detected, compared with 48.7% of participants from FCT. Coinfections were also more commonly detected in Edo State (15.4%) than in FCT (10.0%). Children were more likely to have a pathogen detected than adults (63.1%, 54.8%), with no significant difference in coinfections by site.

High-intensity coinfections (3 or 4 pathogens) were detected in 8 adults and 1 child.

**Figure 1.**
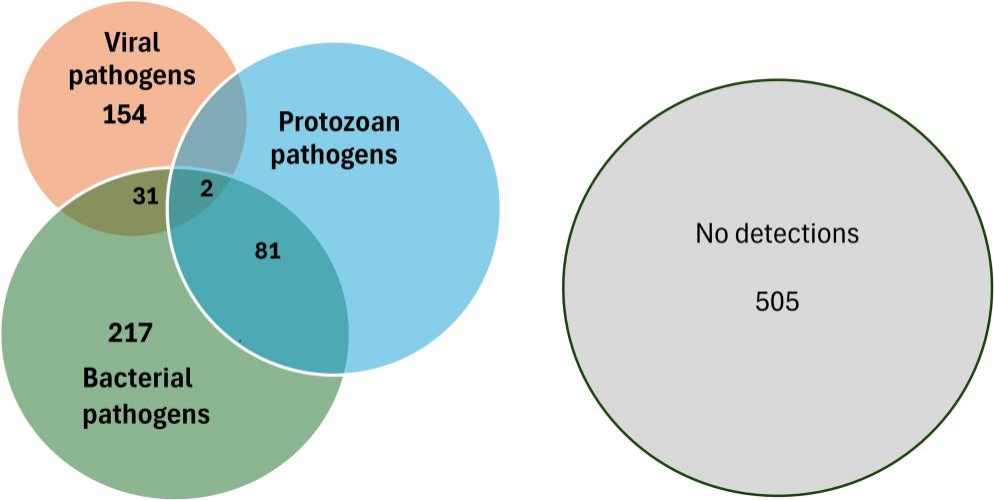
Overlap among pathogen types, indicating the number of single and multiple type codetections among enrolled febrile patients (*N* = 1200)*. *Diagram not to scale.

Bacterial and protozoan pathogens were concomitantly detected among 81 participants (6.8%), bacterial and viral pathogens among 31 participants (2.6%), and viral and protozoan pathogens in 27 participants (2.3%; Figure 1). Two participants were detected with all 3 pathogen types.

The SAFIAN study detected 20 of the 25 pathogens screened. A total of 15 pathogens were detected as a coinfection with another pathogen (Table 3). Three pathogens were detected as single infections only, including *Bartonella* spp., Rift Valley fever virus, and West Nile virus.

**Table 3.** Number and Percentage of SAFIAN Participants With Pathogens Detected, as a Single Infections or Coinfection, by Pathogen

Pathogen	No. of Single Infections (%)	No. of Double Infections (%)	No. of Triple/Quad Infections (%)	Total No. of Infections Detected
RICK	199 (63.8)	106 (34.0)	7 (2.2)	312
PLAS	183 (62.5)	105 (35.8)	5 (1.7)	293
LASV	137 (73.3)	44 (23.5)	6 (3.2)	187
BRUC	2 (14.3)	9 (64.3)	3 (21.4)	14
NMEN	7 (58.3)	4 (33.3)	1 (8.3)	12
DENV	4 (40.0)	4 (40.0)	2 (20.0)	10
ONNV	5 (71.4)	1 (14.3)	1 (14.3)	7
CCHFV	2 (50.0)	2 (50.0)	—	4
CHIKV	1 (16.7)	5 (83.3)	—	6
ZIKV	—	1 (50.0)	1 (50.0)	2
pOPXV	—	2 (100.0)	—	2
pSALM	—	—	1 (100.0)	1
LEPT	—	—	1 (100.0)	1
HEPV	—	1 (100.0)	—	1
CBUR	—	1 (100.0)	—	1
MPOX	—	1 (100.0)	—	1
YPES	—	1 (100.0)	—	1
BART	1 (100.0)	—	—	1
RVFV	1 (100.0)	—	—	1
WNV	1 (100.0)	—	—	1
HTNV	—	—	—	—
LEISH	—	—	—	—
MARV/EBOV	—	—	—	—
TBRU	—	—	—	—
YFV	—	—	—	—
All pathogens	543	287	28	858

Eight pathogens were only found as part of a coinfection, including Zika virus, monkeypox virus, pan-*Orthopoxvirus*, pan-*Salmonella*, hepatitis E virus, *Leptospira* spp., *Coxiella burnetii*, and *Yersinia pestis*. An additional 3 pathogens (chikungunya virus, dengue virus, *Brucella* spp.) were detected where more than half of the cases were coinfected.

The 3 most commonly detected pathogens in the SAFIAN population were *Rickettsia* (*n* = 312), *Plasmodium* (*n* = 293), and Lassa virus (*n* = 184). More than one-third of *Rickettsia* (36.2%) and *Plasmodium* (37.2%) infections were coinfections, along with 26.7% of Lassa virus infections. Additional data on pathogen coinfections are included in the Supplementary Data.

**Figure 2.**
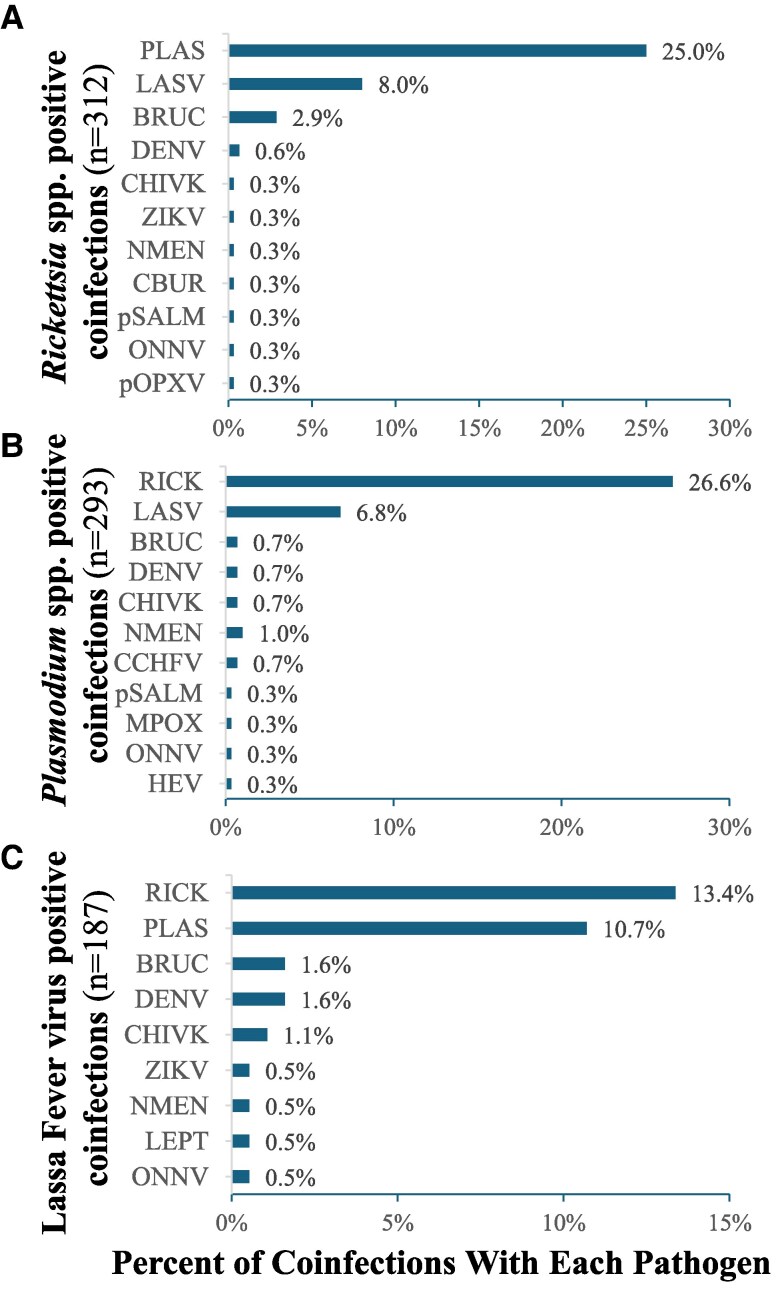
Among participants testing positive for **A**. *Rickettsia* spp., **B**, *Plasmodium* spp, or **C**. Lassa virus, in the SAFIAN study, percent coinfected by each pathogen.

Coinfections identified in the 3 most common pathogens are shown in Figure 2. Most notably, one-quarter of *Rickettsia* and *Plasmodium* cases were coinfected with both pathogens.

### Clinical Outcomes and Symptoms

Overall, few significant differences in patient demographics, symptoms, and clinical outcomes were observed between populations infected with a single pathogen compared with those coinfected with more than 1 pathogen. We examined the 3 most commonly detected pathogens, *Rickettsia*, *Plasmodium*, and Lassa virus. Statistically significant differences for each pathogen infection are presented in Table 4.

**Table 4.** Comparison of Characteristics of Participants Testing Positive for a Single and Multiple Pathogens

*Rickettsia* spp.			
Characteristic	Single Pathogen (*N* = 199)	Coinfections (*N* = 113)	*P* Value
Age (mean [min, max])			
Youth	11.1 (5.0–17.0)	12.0 (5.0–17.0)	.203
Adult	45.4 (18.0–90.0)	38.3 (18.0–82.0)	.004
Cause of fever diagnosis			
Malaria	85 (42.7%)	46 (40.7%)	<.001
Lassa fever	0 (0.0%)	21 (18.6%)	
Typhoid fever	3 (1.5%)	1 (0.9%)	
Other	8 (4.0%)	4 (3.5%)	
Other febrile illness	26 (13.1%)	8 (7.1%)	
No clinical diagnosis	77 (38.7%)	33 (29.2%)	
Treatment			
Antiviral	1 (0.5%)	24 (21.2%)	<.001
Symptoms experienced in the past 7 d			
Vomiting/nausea	47 (23.6%)	41 (36.3%)	.017
Health conditions (self-reported)			
Diabetes	13 (6.5%)	1 (0.9%)	.021

*Plasmodium* spp.			
Characteristics	Single Pathogen (*N* = 183)	Coinfections (*N* = 110)	*P* Value
Season			
Dry season start (15 October–14 November)	24 (13.1%)	8 (7.3%)	.006
Harmattan period (15 November–14 January)	35 (19.1%)	17 (15.5%)	
Dry season end (15 January–15 March)	15 (8.2%)	24 (21.8%)	
Wet season (16 March–14 October)	109 (59.6%)	61 (55.5%)	
Animal contact exposure the month before illness			
Had contact with an animal	73 (39.9%)	30 (27.3%)	.029
Had contact with at least 1 ALIVE animal	71 (38.8%)	29 (26.4%)	.0230
Cause of fever diagnosis			
Malaria	98 (53.6%)	48 (43.6%)	<.001
Lassa fever	0 (0.0%)	15 (13.6%)	
Typhoid fever	3 (1.6%)	2 (1.8%)	
Other	7 (3.8%)	1 (0.9%)	
Other febrile illness	23 (12.6%)	7 (6.4%)	
Sickle cell	1 (0.5%)	0 (0.0%)	
No clinical diagnosis	51 (27.9%)	37 (33.6%)	.357
Treatment			
Antiviral	2 (1.1%)	16 (14.5%)	<.001
Symptoms experienced in the past 7 d			
Unusual/unexplained bleeding	2 (1.1%)	9 (8.2%)	.003

Lassa Virus			
Characteristic	Single Pathogen (*N* = 137)	Coinfections (*N* = 50)	*P* Value
Disposition at discharge			
Discharged to home	132 (96.4%)	44 (88.0%)	.071
Deceased	5 (3.6%)	6 (12.0%)	
Cause of fever diagnosis			
Malaria	5 (3.6%)	4 (8.0%)	.034
Lassa fever	130 (94.9%)	42 (84.0%)	
Other febrile illness	1 (0.7%)	1 (2.0%)	
No clinical diagnosis	1 (0.7%)	3 (6.0%)	
Symptoms experienced in the past 7 d			
Cough	2 (1.5%)	6 (12.0%)	.005

Viral Hemorrhagic Fevers (DENV, CCHFV, LASV, RVFV)			
Characteristic	Single Pathogen (*N* = 144)	Coinfections (*N* = 52)	*P* Value
Season			
Dry season start (15 October–14 November)	2 (1.4%)	4 (7.7%)	.031
Harmattan period (15 November–14 January)	25 (17.4%)	11 (21.2%)	
Dry season end (15 January–15 March)	85 (59.0%)	21 (40.4%)	
Wet season (16 March–14 October)	32 (22.2%)	16 (30.8%)	
Cause of fever diagnosis			
Malaria	8 (5.6%)	5 (10.6%)	.526
Lassa fever	131 (92.3%)	41 (87.2%)	
Other	3 (2.1%)	1 (2.1%)	
No diagnosis	2 (1.4%)	5 (9.6%)	.015
Disposition at discharge			
Discharged to home	138 (95.8%)	45 (86.5%)	.035
Discharged to an alternate care facility	0 (0.0%)	0 (0.0%)	
Deceased	6 (4.2%)	6 (11.5%)	

Additional variables did not demonstrate statistically significant associations and are therefore not shown. Variables reviewed include the following: demographics (age, sex, highest level of education, occupation), recruitment (hospital site, enrollment month and season), health behaviors (number of people in household, bed net use per week, time spent in the bush in past 14 days), animal exposure in the past month (rodent, cat, cow, dog, pig, goat, sheep, chicken, guinea fowl, monkey), severity of illness (duration of fever, current temperature, symptoms, length of stay at hospital), and hospital data (disposition at discharge, hospital assigned cause of fever diagnosis, hospital treatment; self-reported health conditions).

The symptomatic differences were few. *Rickettsia* coinfections had a higher rate of vomiting/nausea, *Plasmodium* coinfections had a higher rate of bleeding, and Lassa virus coinfections had a higher rate of cough. Additional details on coinfections are included in Supplementary Data.

#### Rickettsia

For *Rickettsia*, the mean age for adults was higher among single-pathogen–infected participants than among coinfected participants (45.4 years vs 38.3 years). Participants with *Rickettsia* single-pathogen infections were more likely to be undiagnosed by clinicians (38.7% vs 29.2%). *Rickettsia* coinfected participants were more likely to be diagnosed with Lassa fever virus (18.6% vs 0%) and to be treated with antivirals (21.2% vs 0.5%). Malaria diagnosis was common for both single-pathogen *Rickettsia* (42.7%) and the coinfected group (40.7%). Symptomology was the same in both groups except for the higher rate of vomiting/nausea in the coinfected group (36.3% vs 23.6%). Diabetes was more common in the single-pathogen group (6.5% vs 0.9%) and was the only difference observed of the health conditions.

#### Plasmodium

Participants with *Plasmodium* spp. single-pathogen infections were more likely to be correctly diagnosed with malaria (53.6%) compared with 43.6% of coinfected participants. *Plasmodium c*oinfected participants were more likely to be diagnosed with Lassa fever virus (13.6% vs 0%), more likely to be treated with antivirals (14.5% vs 1.1%) and more likely to be observed with unusual/unexplained bleeding (8.2% vs 1.1%). All other symptoms and all medical conditions were the same between groups. Those with a *Plasmodium* coinfection were more likely to be enrolled in the dry season than those with *Plasmodium* single infection (21.8% vs 8.2%).

#### Lassa Fever Virus

Participants with Lassa fever virus single-pathogen infections were nearly identical to those with coinfections. The only observed difference was presence of a cough in 12% of those coinfected, which affected only 1.5% in the single-pathogen group. All other symptoms, demographics and behaviors were the same.

#### Viral Hemorrhagic Fevers

The group of 4 viral hemorrhagic fevers (dengue virus, Crimean-Congo hemorrhagic fever virus, Lassa virus, and Rift Valley fever virus; *n* = 196) showed a significant difference in seasonality, with more coinfected participants enrolled during the wet season (30.5% of coinfected participants) than the single-pathogen group (22.2%). Additionally, coinfected participants had a greater case fatality rate with 11.5% deceased before discharge compared with only 4.2% of single-pathogen–infected participants. Disposition at discharge was not significantly different for any other groups comparing single infections and coinfections. Coinfected participants more commonly had no diagnosis (9.6% vs 1.4% in the single-pathogen group.)

### Seasonality

Significant differences were observed in the seasonality of coinfections for *Plasmodium*. As shown in Table 4, a greater percentage of *Plasmodium*-positive cases were coinfections during the dry season end (15 January to 15 March; 21.8%) compared to single infections (8.2%). *Plasmodium* coinfections peaked in February (61%) and March (67%) when *Plasmodium* positives were at their lowest point [11]. No significant seasonal differences were observed for coinfections overall or when viewed by pathogen other than *Plasmodium*.

**Figure 3.**
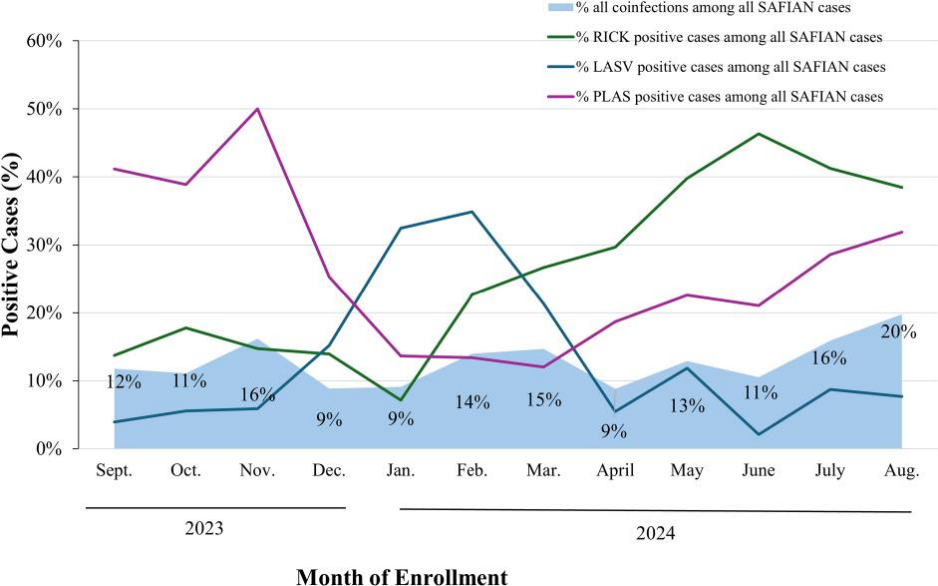
Monthly percent positivity of enrolled SAFIAN participants with any coinfection (light blue), *Rickettsia* spp. (green), *Plasmodium* spp. (purple), or Lassa virus (dark blue). Percentages are based on monthly enrollment numbers.

We reviewed seasonal trends for the 3 most common pathogens detected in the AFI population, which also dominate the coinfections. Seasonal variation was observed in the diversity of the 3 most common pathogens, which coincided with monthly coinfection rates (Figure 3). Although *Plasmodium* and *Rickettsia* shared a similar seasonal distribution, Lassa virus peaked during the dry season of January to March, when the other 2 pathogens experienced a downward trend. The rate of all coinfections showed modest fluctuations by month, ranging from 9% to 20%, but without a consistent trend.

## DISCUSSION

Understanding the full landscape of infectious diseases requires robust multi-pathogen surveillance research, especially in regions where febrile illnesses are common and often misdiagnosed. This study exemplifies that need by screening 1200 febrile patients from 2 tertiary hospitals in Nigeria for 25 different pathogens—spanning bacterial, viral, and protozoan agents. The findings revealed a broad spectrum of 20 circulating pathogens, with coinfections present in 36% of patients where at least 1 pathogen was detected. Strikingly, all coinfections involved either *Plasmodium*, *Rickettsia*, or Lassa fever virus, the 3 most frequently detected pathogens. Without the comprehensive multi-pathogen TAC employed in this study, many of these coinfections might have been misattributed solely to one of these pathogens, significantly underestimating the true diversity of febrile illnesses. These results underscore the critical value of multi-pathogen surveillance in uncovering hidden coinfections and providing a more accurate picture of disease burden in endemic settings.

A danger for coinfected patients in malaria-endemic regions such as Nigeria is the likelihood that pathogens coinfected with *Plasmodium* are overlooked by clinicians because malaria is a common diagnosis in Nigerian hospitals. In the SAFIAN study, this burden is sizable because 72% of all coinfections identified included *Plasmodium*. This further underscores the diagnostic challenge clinicians face, often unaware of the coinfections they may be missing because of limitations in routine testing and undifferentiated symptomology.

The similar nature of febrile symptoms is a key challenge for clinicians, underlying all AFI cases. Although the study captured a detailed symptomology of over 20 symptoms, few significant differences were observed between participants with coinfections and those with a single-pathogen infection. The similarity in presentations may fail to raise clinical suspicion for coinfections, increasing the likelihood that they go undetected during routine evaluation.

Severity of the illness did not differ in coinfected participants, as indicated by length of hospital stay, patient temperature, or death. Our study reported on multiple underlying health conditions, including HIV, sickle cell anemia, and diabetes but did not observe differences in underlying conditions in coinfected populations. Although other studies have noted that coinfected participants have poorer outcomes associated with an immunocompromised state [12], our AFI population did not exhibit this trend. Our unexpected findings highlight the need for further investigation and the development of additional hypotheses tailored to AFI populations.

One finding we observed on health outcomes, among participants infected with 1 of the 4 viral hemorrhagic fevers, those with coinfections exhibited a notably higher case fatality rate (11.5%) than those with single-pathogen infections (4.2%). This suggests that these coinfections may be associated with increased disease severity and poorer clinical outcomes, highlighting the need for comprehensive diagnostic screening and tailored clinical management in febrile patients.

Seasonality may play a role in the appearance of coinfections. Specifically, those with a *Plasmodium* detected during end of the dry season (January to March) exhibited a higher likelihood of coinfection than a *Plasmodium* single infection when compared to other times of the year. Therefore, when *Plasmodium* is identified during the dry season, clinicians may be advised to consider potential coinfections as contributing causes of fever.

Overlapping endemicity and seasonality of multiple pathogens is a challenge in identifying patients with coinfections. For example, malaria, dengue, and chikungunya are often cocirculating and challenge clinical teams in proper diagnosis [7]. Our data show that the prevalence of *Rickettsia* exhibits seasonal patterns analogous to those of malaria, with increased incidence during the wet season. This similar epidemiologic trend between these pathogens has also been observed by Mediannikov et al [13], who remarked on *Rickettsia*'s “silent contribution” to morbidity and mortality caused by febrile illness. Our findings show that 41% of *Rickettsia* coinfections were diagnosed as malaria. A significant concern is the underrecognized presence of *Rickettsia* spp., which has remained undetected in humans in Nigeria, and no diagnostic tests are commonly available [14]. It is imperative that clinicians consider *Rickettsia* in febrile illness diagnoses, particularly during peak malaria transmission periods, to avoid misdiagnosis as malaria and ensure appropriate treatment for *Rickettsia*.

Coinfections within Lassa virus–infected patients are common, although not commonly detected. In a study of suspected Lassa virus samples, Ashcroft et al found that 40% of the 84 samples positive by TAC detected more than 1 target, including mixed bacterial and viral infections, with 4.8% containing 5–7 detected pathogens [15]. Additionally, a study of metagenomic sequencing in Nigeria identified coinfections in confirmed Lassa virus–infected samples [16]. Because accurate PCR laboratory diagnostics are available for Lassa virus, there is a tendency among clinicians to cease further investigation upon a positive identification, potentially overlooking coinfections or alternative pathogens.

This study has several limitations to report. First, the TAC platform used for pathogen detection, while enabling broad molecular screening, is a research use–only tool and is not currently validated for routine clinical diagnosis. The TAC-derived results are not definitive for clinical use but instead highlight potential areas of concern—both clinical and epidemiological—that warrant further investigation. The absence of confirmatory assays for all positive detections limits our ability to definitively attribute clinical illness to detected pathogens. Second, although 25 molecular targets were included in the multi-pathogen testing platform, the SAFIAN study only detected pathogens in half of the participants, leaving a large sample with unidentified cause of febrile illness which could be highlighting either the limitations of the selected panel or the presence of undiagnosed etiologies that require more expansive diagnostic approaches such as metagenomic sequencing. Third, although we examined associations between coinfections and selected clinical outcomes, including mortality, the observational design and sample size were not sufficient to infer causality or to fully adjust for potential confounders such as host immune status or treatment delays. The TAC detection reflects the presence of pathogen genetic material but does not confirm active infection or establish the etiologic cause of fever. Finally, as a hospital-based study conducted in 2 tertiary centers, our findings may not reflect the etiology or clinical profile of febrile illness in community or primary care settings, where diagnostic practices and disease severity may differ. Additionally, as a research use–only detection instrument, the TAC platform requires additional diagnostics for confirmational testing.

Despite the above stated limitations, the study has some considerable strengths. To our knowledge, it represents one of the largest prospective investigations of AFI using multi-pathogen molecular diagnostics in Nigeria. These findings provide novel insights into the burden and complexity of febrile illness in Nigeria and underscore the value of expanded diagnostic capacity for improving patient management and public health preparedness in endemic settings.

## CONCLUSIONS

The findings from this analysis underscore the importance of considering coinfections as potential contributors to febrile illness in patients presenting with undifferentiated fever, especially in areas with limited diagnostic resources. Enhanced clinical awareness and diagnostic vigilance are essential to ensure accurate identification and appropriate management of all underlying pathogens. To facilitate accurate diagnosis, development is needed for clinical tests for pathogens that currently have no nonresearch tests available.

## Supplementary Material

ciaf516_Supplementary_Data

## Supplementary Data

Supplementary materials are available at *Clinical Infectious Diseases* online. Consisting of data provided by the authors to benefit the reader, the posted materials are not copyedited and are the sole responsibility of the authors, so questions or comments should be addressed to the corresponding author.
