# Supplementary material for: High Burden of Coinfections With Epidemic-Prone Pathogens Among Febrile Patients in Nigeria: A Multi-Pathogen Surveillance Study
Source: Clin Infect Dis. 2025 Nov 20;81(Suppl 4):S177–85. doi: 10.1093/cid/ciaf516 (PMC12631764; doi:10.1093/cid/ciaf516)
Supplement: ciaf516_Supplementary_Data [file ciaf516_supplementary_data.zip › Coinfections_Supplemental materials.docx]

# Supplemental Materials

Figures 1 and 2, and Table 1 include additional details on coinfections detected within the SAFIAN population.

Figure 1.

Number of SAFIAN participants with pathogens detected, listed by single infection, double, triple and quadruple pathogen coinfections.

Figure 2.

Number of SAFIAN participants with coinfection detected (double, triple and quadruple), by pathogen

**Table 1.** Number of SAFIAN participants with pathogens detected as a coinfection, by pathogen

| **Pathogen** | **Number of participants coinfected**  **(% of all coinfections)** |
| --- | --- |
| RICK | 113 (74%) |
| PLAS | 109 (72%) |
| LASV | 50 (33%) |
| BRUC | 12 (8%) |
| DENV | 6 (4%) |
| CHIKV | 5 (3%) |
| NMEN | 5 (3%) |
| ZIKV | 2 (1%) |
| pOPXV | 2 (1%) |
| CCHFV | 2 (1%) |
| ONNV | 2 (1%) |
| LEPT | 1 (1%) |
| CBUR | 1 (1%) |
| MPOX | 1 (1%) |
| YPES | 1 (1%) |
| BART | (0%) |
| HEPV | (0%) |
| pSALM | (0%) |
| RVFV | (0%) |
| WNV | (0%) |
