# Supplementary figures and images for: High Burden of Coinfections With Epidemic-Prone Pathogens Among Febrile Patients in Nigeria: A Multi-Pathogen Surveillance Study
Source: Clin Infect Dis. 2025 Nov 20;81(Suppl 4):S177–85. doi: 10.1093/cid/ciaf516 (PMC12631764; doi:10.1093/cid/ciaf516)

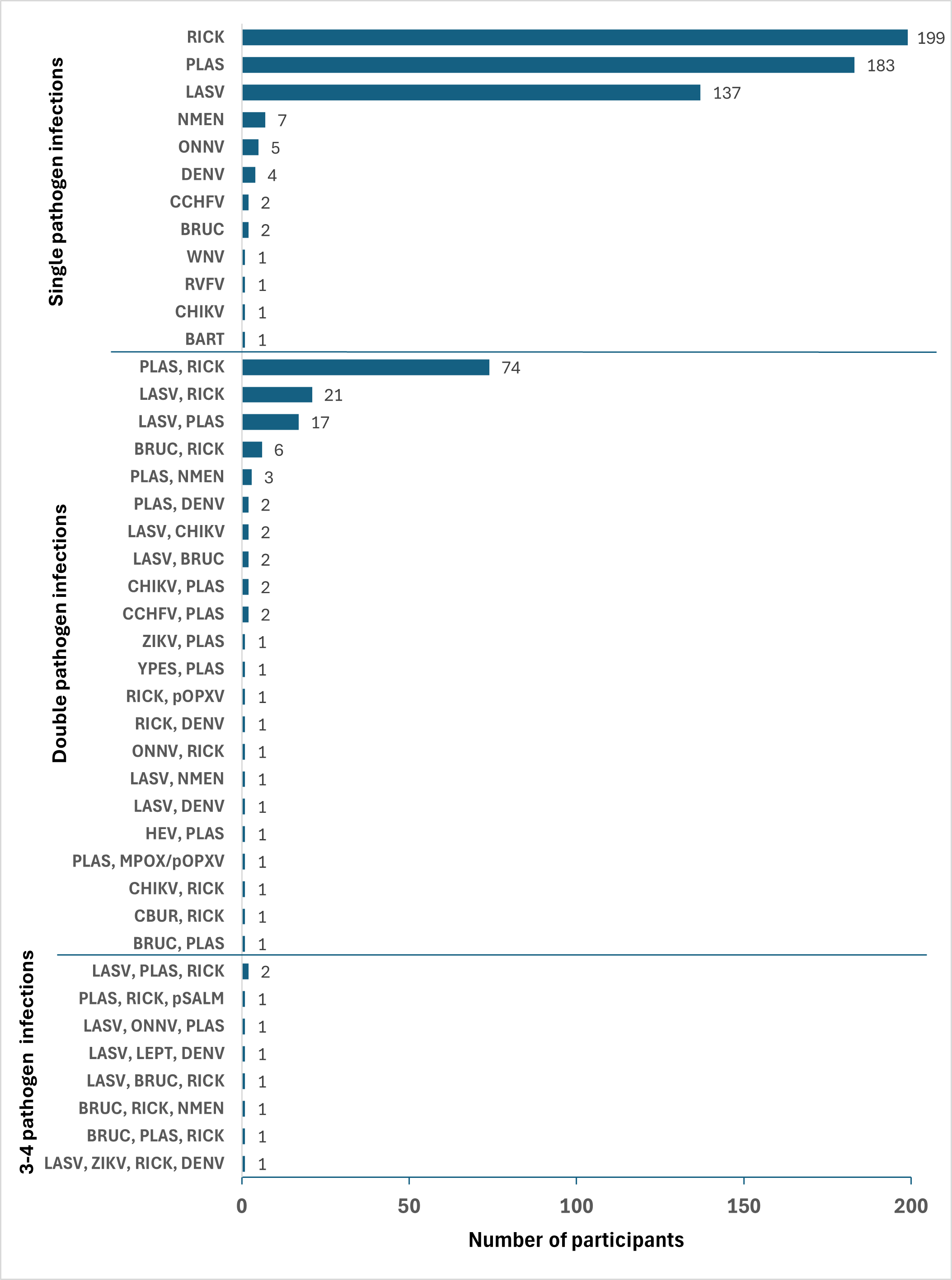

Supplement: ciaf516_Supplementary_Data [file ciaf516_supplementary_data.zip › Figure1_supplemental-v2.tif]

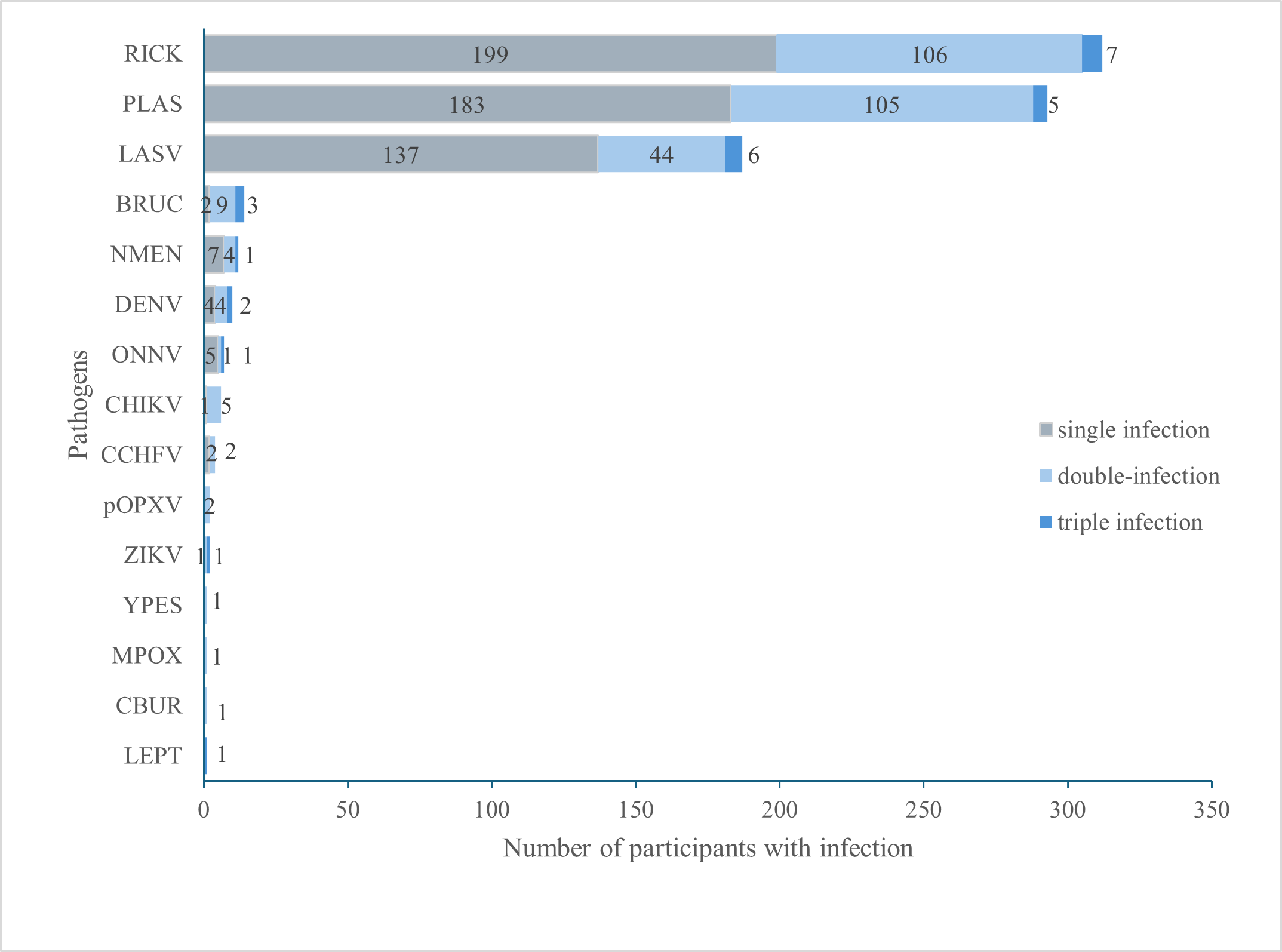

Supplement: ciaf516_Supplementary_Data [file ciaf516_supplementary_data.zip › Figure2_Supplemental material.tif]
